# Drivers of antibiotic prescribing in children and adolescents with febrile lower respiratory tract infections

**DOI:** 10.1371/journal.pone.0185197

**Published:** 2017-09-28

**Authors:** Verena Gotta, Philipp Baumann, Nicole Ritz, Aline Fuchs, Gurli Baer, Jessica M. Bonhoeffer, Ulrich Heininger, Gabor Szinnai, Jan Bonhoeffer, for the ProPAED study group

**Affiliations:** 1 Department of Pediatric Pharmacology and Pharmacometrics, University of Basel Children’s Hospital, Basel, Switzerland; 2 Department of Pediatric Infectious Diseases and Vaccinology, University of Basel Children’s Hospital, Basel, Switzerland; 3 University of Basel Children’s Hospital, Basel, Switzerland; 4 Department of Pediatric Endocrinology and Diabetology, University of Basel Children’s Hospital, Basel, Switzerland; TNO, NETHERLANDS

## Abstract

**Background:**

Knowledge of key drivers for antibiotic prescribing in pediatric lower respiratory tract infection (LRTI) could support rational antibiotic use. Thus, we aimed to determine the impact of clinical and laboratory factors on antibiotic prescribing in children and adolescents with febrile LRTI.

**Methods:**

Pediatric patients from the standard care control group of a randomized controlled trial (ProPAED) investigating procalcitonin guided antibiotic treatment in febrile LRTI were included in a multivariate logistic regression analysis to evaluate the impact of laboratory and clinical factors on antibiotic prescribing.

**Results:**

The standard care control group of the ProPAED study comprised 165 LRTI patients (median age: 2.7 years, range: 0.1–16), out of which 88 (55%) received antibiotic treatment. Factors significantly associated with antibiotic prescribing in patients with complete clinical and laboratory documentation (n = 158) were C-reactive protein (OR 5.8 for a 10-fold increase, 95%CI 2.2–14.9), white blood count beyond age-dependent reference range (OR 3.9, 95%CI 1.4–11.4), body temperature (OR 1.7 for an increase by 1°C, 95%CI 1.02–2.68), and pleuritic pain (OR 2.8, 95%CI 1.1–7.6). Dyspnea (OR 0.3, 95%CI 0.1–0.7) and wheezing (OR 0.3, 95%CI 0.13–0.95) were inversely associated with antibiotic prescribing.

**Conclusion:**

Laboratory markers were strong drivers of antibiotic prescribing in children with febrile lower respiratory tract infections, in spite of their known poor prediction of antibiotic need. Building on current guidelines for antibiotic treatment in children with febrile LRTI, a reliable decision algorithm for safe antibiotic withholding considering the laboratory and clinical factors evaluated in this study has the potential to further reduce antibiotic prescribing.

## Introduction

Lower respiratory tract infections (LRTI) bear high mortality and morbidity in children and adolescents with community acquired pneumonia (CAP) being the leading cause of death worldwide with 1.3 million fatal pediatric cases each year [1]. Further, LRTI are associated with non-fatal complications like pleural effusions, empyema (up to 12% of pneumonia cases) or necrotizing pneumonia (0.8%) which are feared by both hospital and general physicians [2, 3]. However, identifying patients benefitting from antibiotic treatment among those not in need remains a major diagnostic challenge. Thus, physicians tend to err on the safe side and overuse antibiotics according to varied approaches as reflected in the different antibiotic prescription rates across countries and institutions [4–8]. A better understanding of the decision to prescribe or withhold antibiotics is crucial for future restrictive treatment strategies. Factors leading to antibiotic prescription are known to be complex [5] and are scarcely elucidated in the pediatric population. Given that adult studies have identified e.g. a strong impact of C-reactive protein (CRP) (odds ratio 98.1 for exceeding 50 mg/L) on physicians`decision to prescribe antibiotic medication for LRTI [9] there is a need for clarification of what sways the decision to prescribe antibiotics for LRTI in children and adolescents. Thus, we aimed at characterizing the laboratory and clinical drivers of antibiotic prescribing for pediatric LRTI with a subanalysis of the ProPAED trial [10]. This study was among the first randomized controlled trials in children evaluating the feasibility and effect of Procalcitonin (PCT) guided treatment for respiratory tract infections [11, 12] after first observational studies suggested that PCT might be considered in pediatrics. The ProPAED investigators tested established adult PCT cut-off ranges in a cohort of pediatric emergency departments of tertiary care centers [13–19].

## Methods

### Study population

This is a hypothesis generating post-hoc analysis from a randomized controlled trial (ProPAED) evaluating PCT as a biomarker to reduce antibiotic treatment of LRTI in children and adolescents [10]. Briefly, children and adolescents 1 month to 18 years of age presenting with LRTI to the pediatric emergency departments of two pediatric tertiary care centers in Switzerland between 01/2009 and 02/2010 were included. Acute LRTI was defined by the presence of fever (≥38°C measured in the hospital at study enrolment or at home), and at least one of the following symptoms: tachypnea, dyspnea, wheezing, late inspiratory crackles, bronchial breathing, and/or pleural rub. CAP was defined as febrile LRTI with a new or increasing alveolar infiltrate on chest radiograph. In the original ProPAED trial patients were randomized to the PCT guided intervention group or to the standard care control group with treatment based on international guidelines [4]. For the analysis of factors associated with antibiotic prescribing only patients from the standard care control group were included. Follow-up was available for 98% of all ProPAED patients and no increase of adverse events was associated with antibiotic withholding in either of the treatment groups [10].

### Variables

The following variables were included for analysis. As clinical parameters presence or absence of pleuritic pain, pleural rub, wheezing, tachypnea, dyspnea, late inspiratory crackles, reduced breathing sound, bronchial breathing, body temperature, and heart and respiratory rate were chosen. For demographic variables and patient history age, pneumococcal and *Haemophilus influenzae* type b vaccination status (as classified according to S1 and S2 Tables), and days of fever preceding presentation at hospital were included. As laboratory and microbiology parameters CRP, and white blood cell count (WBC), respiratory syncytial virus (RSV) type A/B or influenza A/B on nasopharyngeal aspirate (NPA) were included. All laboratory and microbiological tests were performed at study enrolment. Blood culture results were not included in this analysis since they were not systematically obtained and therefore available only in 72 (43%) of patients with 4 positive results.

### Definitions

WBC was classified as normal or beyond age-dependent reference range according to reference values provided by the hospital-specific laboratory (S3 and S4 Tables). Heart rate was classified as normal or elevated according to age-dependent references ranges (S5 Table) [20], respiratory rate was classified as elevated if exceeding WHO age-dependent reference ranges for children up to 5 years of age, and if exceeding percentile 90 as reported by Fleming et al. for children >5 years of age (S6 Table) [21].

### Statistical analysis

#### Analysis of factors associated with antibiotic prescription

The association of clinical, demographic and laboratory variables with antibiotic prescribing was assessed using exploratory graphical evaluation and univariate logistic regression analysis (S1 Fig). For variables showing a significant association, sensitivity, specificity, accuracy and area under the receiver operating characteristics (ROC) curve were calculated as performance measures to predict or classify antibiotic prescribing. For continuous variables these performance measures were calculated for the best threshold (Youden index) which was determined by ROC analysis. Subsequently, variables showing a significant association in univariate analysis were included in a multivariate logistic regression model, followed by stepwise backward deletion of non-significant variables. The predictive performance of the final multivariate model was expressed regarding accuracy, sensitivity and specificity (using a probability threshold of ≥0.5 for classification of antibiotic prescribing). Continuous variables were partly transformed (e.g. log-transformation, categorization) and centered to normal values (instead of zero) for regression analysis to linearize relationships, according to clinical relevance and graphical evaluation of the appropriateness of assumed relationships (S2–S5 Figs). Missing data was ignored (complete-case analysis), other than vaccination status and NPA test results, where missingness was interpreted as incomplete vaccination status or negative NPA test result, respectively. The global goodness of fit of the final multivariate model was assessed by unweighted sum of squares statistic (Cessie, van Houwelingen, Copas, Hosmer unweighted sum of squares test) and residual diagnostics (Pearson residuals were plotted and any points > |3 standard deviations| were investigated); overdispersion was assessed and deemed not to be present if the scale parameter (estimate of the residual mean square) was within the interval [0.85, 1.15]. The relative goodness of fit compared to simpler models following single term deletion was assessed using the likelihood-ratio test.

#### Sensitivity analyses

The following three sensitivity analyses were performed: (1) The interaction between pleuritic pain and age (stratified by 2 years of age), was assessed, since pain is difficult to be assessed children < 2 years. (2) Elastic net regression was used to verify robustness of selected covariates. (3) Remaining missing variables (<3%) were multiply imputed by fully conditional specification using the R package mice and re-tested in the logistic regression analysis [22]. All statistical analyses were performed in R (version 3.2.1; R Development Core Team, Vienna, Austria, http://www.r-project.org). Example codes of the main analyses can be found in the supplemental data (S1 Text).

### Ethics

Both the ethics committee of the University of Basel and Kanton Aargau approved the trial protocol. Written informed consent was obtained from all patients or their care takers. The trial was registered with the International Standard Randomized Controlled Trial Number Register (ISRCTN 17057980).

## Results

### Factors associated with antibiotic prescription

Out of 169 patients in the standard care group, a total of 165 patients with complete data sets for the current analyses were included. Of these, 93 (56%) received antibiotic treatment (Table 1). The majority of patients treated with an antibiotic (n = 78, i.e. 84%) received it on the first day of study inclusion.

**Table 1:** Patients` characteristics.

	Antibiotic treatment	No antibiotic treatment
**Total number of patients** [n (%)]	93	72
received antibiotic at day 1	78 (84)	NA
received antibiotic > day 1	15 (16)	NA
antibiotic pretreatment	16 (17)	1 (1)
**Clinical diagnosis** (at inclusion) [n (%)]		
Bronchitis/Bronchiolitis	10 (11)	50 (69)
Bronchitis/Bronchiolitis + Pneumonia	24 (26)	17 (24)
Pneumonia	59 (63)	5 (7)
**Radiologic diagnosis** [n (%)]		
Bronchitis/Bronchiolitis	23 (25)	41 (57)
Bronchopneumonia	49 (53)	11 (15)
Lobar pneumonia	17 (18)	1 (1)
Radiography not performed	4 (4)	19 (26)
**Age in years** [median (range)]	3.9 (0.1–15.3)	1.9 (0.1–15.5)
< 5 years [n (%)]	57 (61.3)	60 (83.3)
< 2 years [n (%)]	26 (28.0)	39 (54.2)
**Temperature** [°C (range)]	38.6 (36.3–40.7), NA = 1	38.2 (35.8–39.9)
**Preceding days of fever** [days (range)]	3 (1–14), NA = 2	2 (1–12)
**Heart rate** [1/min (range)]	138 (84–192), NA = 3	141 (88–193), NA = 2
**Respiratory rate** [1/min (range)]	38 (16–80), NA = 2	40 (18–80), NA = 3
**C-reactive protein** [mg/L (range)]	37 (3–653), NA = 3	9 (3–144), NA = 1
**White blood cell count** [G/L (range)]	13.4 (3.3–54.8), NA = 3	9.5 (4.4–24.4)
beyond reference range [n (%)]	46 (50) 6 low, 40 elevated	9 (12) 4 low, 5 elevated
normal [n (%)]	44 (40)	63 (88)
**Symptoms at inclusion [n (%)]**		
Wheezing	9 (10)	37 (51)
Dyspnea	47 (51	57 (79)
Reduced breathing sound	37 (40)	10 (14)
Pleuritic pain	36 (39)	15 (21)
Crackles	20 (22)	24 (33)
Bronchial breathing	16 (17)	7 (10)
Late inspiratory crackles	42 (45)	25 (35)
Pleural rub	2 (2)	0
**Vaccination status** [n (%)]		
Pneumococcal vaccination		
Incomplete or unknown	68 (73, NA = 10)	36 (50, NA = 6)
*Haemophilus influenzae* type b vaccination		
Incomplete or unknown	24 (26, NA = 9)	18 (25, NA = 3)
**Nasopharyngeal aspirate** [n (%)]		
Negative or not performed	40 (43, NA = 5)	29 (40, NA = 5)
Human metapneumovirus	13 (14)	5 (7)
Respiratory syncytial virus A/B	7/6 (14)	18/6 (33)
Influenza A /B	5/1 (6)	3/1 (6)
H1N1	5	2
P1V1	1	2
*Mycoplasma pneumoniae*	7	1
*Chlamydophilia pneumoniae*	1	-
Adenovirus	1	1
Combinations	6	4
**Hospitalization** [n (%)]	61 (66)	34 (47)
**Education** (mother) [n (%)]		
School	15 (17)	21 (30)
Apprenticeship	48 (54)	30 (43)
Technical college	14 (16)	8 (11)
University	12 (13)	11 (16)

NA: not applicable

**Fig 1:**
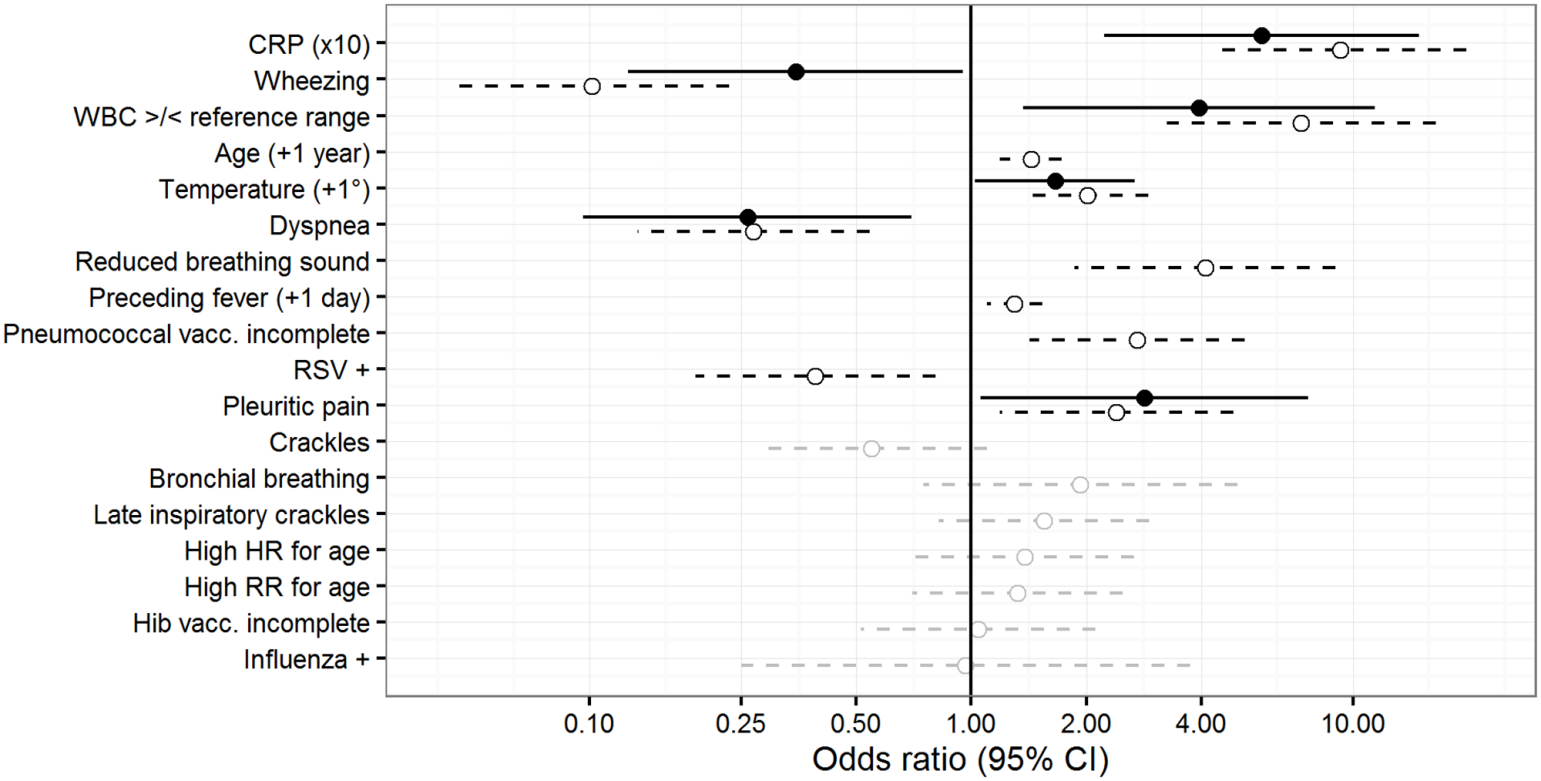
Associations of laboratory and clinical factors with antibiotic prescribing. Odds ratio estimates with 95% confidence intervals (95%CI) from univariate and multivariate logistic regression analysis. Open circles and dashed lines: univariate OR and 95%CI; variables are presented in decreasing order of strength of association with antibiotic prescription (p-value, details: see Table 2), grey circles indicate a non-significant association (p-value ≥ 0.05), the OR estimate for pleural rub (OR>>10 with 95%CI ranging from 0-infinity, Table 1) is not illustrated. Black dots and solid lines: multivariate OR with 95%CI of variables remaining after backward deletion of non-significant variables. Vertical line: OR = 1 indicating no association with antibiotic prescription, ORs > 1 indicate an association with increased antibiotic prescription, ORs < 1 indicate an association with reduced antibiotic use. CRP: C-reactive protein. WBC: White blood cell count. Vacc.: Vaccine. RSV: Respiratory syncytial virus. HR: Heart rate. Hib: *Haemophilus influenzae* type b.

Eleven of 19 assessed clinical and laboratory variables showed a significant association with antibiotic prescribing (Table 2). Of these, eight variables were associated with increased antibiotic prescription and three with reduced antibiotic prescription. Corresponding odds ratio estimates from logistic regression analyses are summarized in Table 2 for all tested clinical and laboratory variables and illustrated in Fig 1 together with the respective multivariate estimates. See complete sensitivity for univariate regression analysis in S7 Table.

**Table 2:** Variables associated with antibiotic prescribing in univariate logistic regression.

Variable (definition of baseline)	Baseline antibiotic treatment % (95%CI)	Univariate OR (95%CI)	Definition of variable effect	Univariate p-value
**C-reactive protein** (10 mg/L)	40 (31–50)	9.3 (4.3–19.8)	for a 10 fold increase	9.2 x 10^−9^
**Wheezing** (absence)	71 (62–78)	0.1 (0.0–0.2)	presence of symptom	6.1 x 10^−8^
**WBC** (normal for age)	41 (32–51)	7.3 (3.3–16.5)	> or < age-dependent reference range	1.5 x 10^−6^
**Age** (0 years)	32 (20–46)	1.4 (1.2–1.7)	for each additional year of age until 5 years	0.0002
**Temperature** (37°C)	34 (22–47)	2.0 (1.4–2.9)	for each additional degree (in °C)	0.0002
**Dyspnea** (absence)	75 (63–85)	0.3 (0.1–0.5)	presence of symptom	0.0002
**Reduced breathing sound** (absence)	47 (39–56)	4.1 (1.9–9.0)	presence of symptom	0.0004
**Pneumococcal vaccination** (complete for age)	41 (29–54)	2.7 (1.4–5.2)	Incomplete or unknown	0.0026
**Preceding days of fever** (1 day)	44 (32–54)	1.3 (1.1–1.5)	for each additional preceding day of fever	0.0029
**Respiratory syncytial virus +** (negative or unknown NPA test)	62 (53–70)	0.4 (0.2–0.8)	positive NPA test	0.011
**Pleuritic pain** (absence)	50 (41–59)	2.4 (1.2–4.9)	presence of symptom	0.015
**Crackles** (absence)	60 (51–69)	0.5 (0.3–1.1)	presence of symptom	0.090
**Bronchial breathing** (absence)	54 (46–62)	1.9 (0.7–5.0)	presence of symptom	0.17
**Late inspiratory crackles** (absence of symptom)	52 (42–62)	1.5 (0.8–2.9)	presence of symptom	0.18
**Heart rate** (normal for age)	51 (38–64)	1.4 (0.7–2.7)	High for age	0.34
**Respiratory rate** (normal for age)	53 (41–64)	1.3 (0.7–2.5)	High for age	0.39
**Hib vaccination** (complete for age)	56 (47–65)	1.0 (0.5–2.1)	Incomplete or unknown	0.91
**Influenzae** (negative or unknown NPA test)	56 (49–64)	1.0 (0.2–3.7)	positive NPA test	0.96
**Pleural rub** (absence)	56 (48–63)	>10.0 (0-infinity)	presence of symptom	0.99

Number of patients included in univariate analysis: n = 165, besides for C-reactive protein: n = 161, temperature: n = 164, heart rate: n = 160, respiratory rate: n = 160, and WBC: n = 16. NPA: nasopharyngeal aspirate, OR: odds ratio, WBC: white blood cell count. p-values were used to order variables in decreasing order of statistical significance.

**Fig 2:**
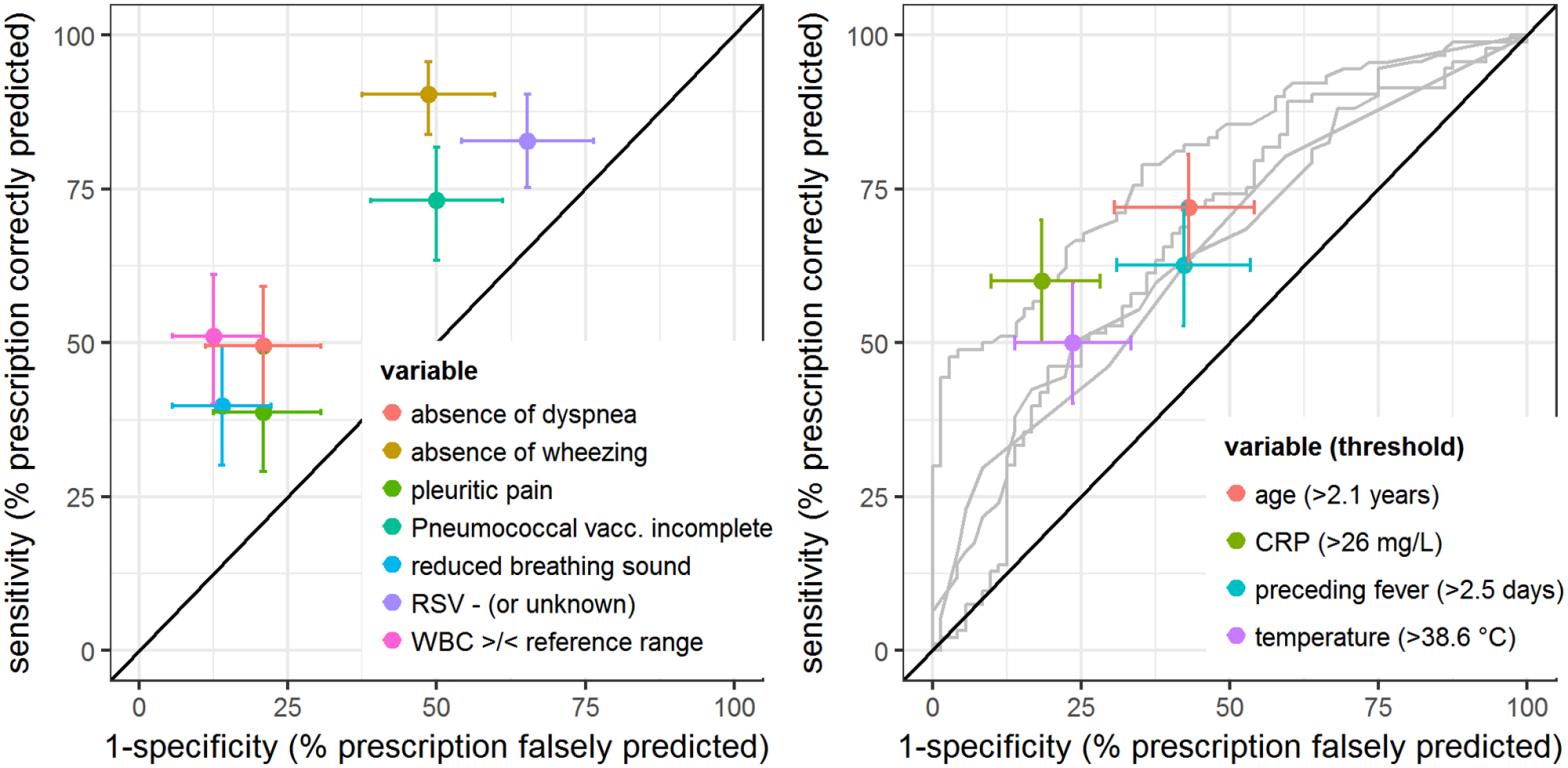
Sensitivities and specificities for associations with antibiotic prescribing. Illustration of sensitivities and specificities (dots) with 95% confidence intervals (crosses) of each single variable for antibiotic prescription. Left panel: dichotomous variables. Right panel: For continuous variables the range of sensitivities and specificities for all possible thresholds is illustrated (gray receiver operating characteristic curves), as well as the sensitivity and specificity associated with the best threshold estimates. Best threshold estimates (95%CI) were: CRP: 26 (12–55) mg/L, age: 2.1 (1.1–4.8) years, temperature: 38.6 (37.3–38.9) °C, preceding fever: 2.5 (1.5–5.5) days. CRP: C-reactive protein. RSV: Respiratory syncytial virus. WBC: White blood cell count.

Fig 2 presents sensitivities and specificities for all eleven variables. The highest sensitivity was calculated for the absence of the symptom wheezing (90%). However, specificity was low (51%). Best specificity (92%) was calculated for a normal white blood cell count. However, here sensitivity was low (50%).

For multivariate logistic regression analysis: 158 patients with complete clinical and laboratory findings of all variables were included (Table 3). Of those, 87 (55%) received an antibiotic. Six of the initially eleven significant variables were retained in this final model, after step-wise backwards-deletion of non-significant associations. Model diagnostics of goodness of fit and dispersion did not indicate model misspecification, or preference of simpler models. Multi- and univariate odds ratio estimates are contrasted in Fig 1.

**Table 3:** Variables associated with antibiotic prescription in multivariate logistic regression.

Variable (definition of baseline^a^)	Effect^b^ (multivariate OR)	Definition of effect	p-value
**C-reactive protein** (10 mg/L)	5.8 (2.2–14.9)	for a 10 fold increase	0.0003
**Dyspnea** (absence of symptom)	0.3 (0.1–0.7)	presence of symptom	0.008
**WBC** (normal)	3.9 (1.4–11.4)	> or < age-dependent reference range	0.011
**Temperature** (37°C)	1.7 (1.0–2.7)	for each additional degree (in °C)	0.040
**Pleuritic pain** (absence of symptom)	2.8 (1.1–7.6)	presence of symptom	0.038
**Wheezing** (absence)	0.3 (0.1–0.9)	presence of symptom	0.039

^a^Baseline probability estimate: 40% antibiotic prescription (20–64%).

^b^Variables removed after stepwise backward deletion of non-significant associations (OR and p-values from full multivariate model): age (OR = 0.97, p = 0.84), reduced breathing sound (OR = 1.08, p = 0.89), incomplete pneumococcal vaccination status (OR = 0.94, p = 0.91), positive RSV test (OR = 0.51, p = 0.20), days of fever (OR = 1.20, p = 0.08). OR: Odds ratio. WBC: White blood cell count.

The multivariate model explained the antibiotic prescribing in 126 patients (accuracy: 80%, 95%CI: 73–86%), with a correct prediction of antibiotic prescription of 83% (sensitivity, 95%CI: 76–86%) and a correct prediction of withholding antibiotic therapy of 76% (specificity; 95%CI: 69–82%). Characteristics of falsely predicted patients are summarized in S8 Table.

### Sensitivity analyses

Subgroup analysis indicated that the association of pleuritic pain with antibiotic prescribing was reduced in younger children (in < 2 years OR = 1.1, p = 0.896, n = 65) compared to the OR in the full analysis (OR = 2.4, p = 0.015, n = 165), and older children only (OR = 2.7, p = 0.037 in patients > = 2 years, n = 100); including a formal interaction term for this age-dependent association reached however not statistical significance of pleuritic pain in neither group. Elastic net regression suggested lowest misclassification error (optimal regularization parameter lambda from 100-fold cross-validation) when 9 covariates are included in a multivariate model (CRP, wheezing, WBC, temperature, dyspnea, preceding days of fever, RSV+, pleuritic pain, crackles). A simpler/more regulized model with only 4 covariates (CRP, Wheezing, WBC, dyspnea) could be favored, when accepting a larger error, within one standard error of the minimum. Using a higher penalty (alpha = 1, i.e. lasso) for covariable selection, the same 4 covariates would be selected (CRP, wheezing, WBC, dyspnea) to obtain lowest misclassification error.

Results of univariate regression analysis did not significantly change when estimating coefficients from multiply imputed datasets (the same 11 variables showed significant associations with antibiotic prescribing with a p-value < 0.05). The multivariate regression results changed slightly, with pleuritic pain removed in backward deletion due to a p-value of 0.056 (compared to p = 0.038 in the initial analysis); all other 5 variables (CRP, wheezing, WBC, dyspnea, temperature) were retained with a p-value < 0.05.

## Discussion

This hypothesis generating study investigated associations of laboratory and clinical findings with antibiotic prescription in children and adolescents with LRTI presenting to the pediatric emergency department. The main finding of this study was the strong association of elevated routine laboratory parameters (CRP and WBC) with increased antibiotic prescribing. Four clinical factors (dyspnea, body temperature, pleuritic pain and wheezing) were also associated with antibiotic prescribing and explained, together with the laboratory parameters, 80% of the antibiotic prescription behavior.

### Laboratory parameters associated with increased antibiotic prescription

Although the diagnostic utility of the laboratory values is known to be limited, in this study they have been found to play a major role for antibiotic prescribing. The strongest association with prescription of antimicrobial agents was found in the multivariate analysis for higher than normal CRP. CRP has been shown in the past to have low predictive values as a single and combined biomarker for LRTI in need of antimicrobial treatment [23–25]. In addition, sensitivities and specificities for differentiation between suspected viral and bacterial LRTI have been revealed to be highly variable on several occasions [23, 24, 26–28]. Pediatric data on the impact of CRP on antibiotic prescribing for LRTI is not available to the best of our knowledge. A study in adults, however, showed a CRP exceeding 50 mg/L to be a strong driver (OR of 98.1) for antimicrobial prescription, consistent with our results [9]. Along the same lines evidence on CRP-guided reduction of antibiotic use in adult LRTI is emerging with a recent meta-analysis showing a moderate reduction of antibiotic prescription (ORs 0.30–0.73) when CRP thresholds are included in treatment decision making [29]. The second strongest association with increased antibiotic prescription was found for an elevated WBC count. This is somewhat surprising as weak predictive performance for WBC as single and combined laboratory parameter in pediatric LRTI is well known [26–28, 30–33]. This may be explained by older pediatric data showing WBC count >15 G/L to be predictive of a good response to antimicrobial treatment in pediatric pneumonia [34]. However, this study of the 1970s did not etiologically evaluate viral or mycoplasma species. Modern studies assessing a broader etiologic spectrum showed that the nasopharyngeal presence of *Mycoplasma pneumoniae*, influenza virus, or adenovirus is associated with elevated WBC as well, blurring its diagnostic performance in LRTI [31–33].

In the ProPAED study, physicians were encouraged to order WBC and CRP at study enrolment in all age groups. This might have introduced bias, as laboratory evaluation is not routinely necessary in young children. However, including only patients > = 2 years of age in a subanalysis did not change the result: CRP and WBC remained significantly associated with antibiotic prescribing (CRP: OR = 7.3 vs 9.3 in full analysis; WBC; OR = 6.3 vs 7.3, (all p-values < 0.01)).

### Clinical parameters associated with increased antibiotic prescription

Clinical parameters predictive for antibiotic prescribing were pleuritic pain and body temperature. Pleuritic pain may be associated with more severe forms of pneumonia particularly with exudative pleural effusions or empyema, for which hospitalization and intravenous antibiotic treatment is required. However, previous studies have shown that sensitivity of pleuritic pain for severe pneumonic infection in young children is limited as children with any LRTI or non-complicated CAP can present with chest or abdominal pain without evidence of effusions or empyema [4, 35]. Further, young children may not be able to adequately locate pleuritic pain and they might rather state non-specific abdominal pain instead. Toddlers may not be able to communicate mild pleuritic pain as a symptom. Accordingly, in the sensitivity analysis, pleuritic pain lost its association with antibiotic prescribing in children younger than 2 years. In the present study population, only five of 48 patients with pleuritic pain had unilateral pleural effusions determined by expert chest radiograph review, none of them required drainage. An underestimation of the clinical sign pleuritic pain in young children cannot be excluded.

Body temperature has been shown to be a predictor of antibiotic prescribing in a large Italian study including all kinds of respiratory tract infections in pediatric primary care setting. Fever >38°C resulted in a two-fold chance of being treated with antibiotics (OR 2.34; 95%CI 1.97–2.79) [5], which is in line with our findings. Fever is included in CAP definitions and reassessment algorithms triggering thorough examination, as persistent fever may indicate development of complications and complications can predict prolonged fever [36, 37]. However, fever is common in children with LRTI on first presentation (88–96%) and non-specific to viral or bacterial etiology and thus need for antibiotic prescription [38].

Tachypnea is included in WHO, Infectious Diseases Society of America (IDSA) and British Thoracic Society (BTS) guidelines as a clinical sign for severity of LRTI disease [2, 4, 39]. It is an easily accessible clinical feature suitable for initial and follow-up evaluation of LRTI. The present analysis failed in univariate regression analysis to show tachypnea as a predictor of antibiotic prescribing. One explanation is that tachypnea as a sign of LRTI is observed in both, viral LRTI and bacterial LRTI [40]. Another explanation might be that clinicians rarely consider this parameter and more likely rely on oxygen saturation.

Incomplete pneumococcal vaccination status was associated with increased antibiotic prescription in univariate regression analyses, but not in the multivariate model. However, incomplete vaccination might drive physicians to suspect pneumococcal infection in those not vaccinated, and this may influence their prescription behavior. It may be well possible, that incomplete vaccination status might be associated with antibiotic use in future studies.

### Clinical parameters associated with decreased antibiotic prescription

Wheezing and dyspnea were independently associated with lower rates of antibiotic prescribing: Particularly wheezing is associated mostly with viral infections, which is also reflected by international guidelines where antibiotic treatment is not recommended for children with mild fever, wheeze, and signs of upper respiratory tract infections [2, 4].

In multivariate analysis, the combination of six factors including CRP, dyspnea, WBC, body temperature, pleuritic pain and wheezing explained 80% of antibiotic prescribing and resulted in more than half of the patients presenting with LRTI to the emergency departments being treated with antibiotics. Importantly the present study demonstrates that there may be an opportunity for further reduction of antibiotic prescribing for febrile LRTI based on the availability of improved biomarkers and clinical parameters. Although some clinical symptoms did not show a statistically significant association with antibiotic prescription in our study (like bronchial breathing, late inspiratory crackles, elevated heart/respiratory rate, pleural rub, all with OR point estimates < 2.0), their consideration in future larger studies could still be of advantage: with our sample size we calculate a power of only < 60% to detect such a significant univariate OR ≤2.

One limitation of the study is that the impact of chest radiographs on antibiotic prescribing could not be assessed due to potential selection bias. Chest radiographs were not mandatory for the initial evaluation during the ProPAED study resulting in chest radiographs being performed in only 87% of all ProPAED patients. However, a background re-assessment of the chest radiographs based on the WHO standard [41] showed a 66% diagnostic accordance with the physicians’ emergency room diagnosis (S9 Table). Inter-observer agreement in the evaluation of chest radiographs in previous studies has been shown to be highly variable: depending on the study setting between 20 to 91% [42–44]. While chest radiographs were not included in the regression model, this background analysis alone demonstrates the poor accordance of chest radiographs with clinical diagnosis and consecutive treatment.

A limitation of the original ProPAED trial with a potential impact on this analysis is the treatment of the intervention group and the standard care control group by the same emergency department team. First, a Hawthorne effect for being under study observation and second, a spill-over effect for learning from the rather restrictive PCT guidance intervention group cannot not be excluded. Especially, as the antibiotic prescription rate was indeed lower in the ProPAED control group compared to historical controls from the same center [10]. The fact of being part in a prospective study with the aim to reduce antibiotic prescription might be an independent factor driving physicians to decreased antibiotic prescription.

A further limitation may be that interactions between patients, parents and physicians were not included as endpoints in the original ProPAED study. Thus, these variables could not be included in the regression analysis. So far, large-scale studies have provided valuable insights into non-clinical factors influencing physicians when making decisions on LRTI treatment. Ahmed et al. showed that pediatric patients are at lowest risk for antibiotic exposure when visiting pediatricians in contrast to emergency department doctors or family practitioners, as the antibiotic prescribing rate in pediatricians is one-third of the rate in emergency department staff [6]. Moro et al. showed that the physicians’ perception of parents expectations to receive antibiotic treatment for their child’s LRTI was a strong driver for antibiotic prescribing (OR 12.8), even when this perception was wrong, as revealed in parents interviews before consultation [5]. These studies have been performed in general practice and influence of parental treatment expectations remain to be elucidated in the tertiary care setting.

Our model did explain 80% of decisions to prescribe antibiotics but leaves 20% unexplained. Several large studies as well as the latest meta-analysis of Lucas et al. demonstrated that a main factor driving antibiotic prescribing in pediatric LRTI is diagnostic uncertainty [5, 45, 46]. However, we have not assessed diagnostic uncertainty in the prescribing physician. As this may be a significant factor also in tertiary care decision making, we suggest quantification of this impact in further research.

## Conclusions

CRP and WBC were strong drivers of antibiotic prescribing in children with febrile LRTI, in spite of their known poor predictive value for antibiotic requirement. In contrast, antibiotics were used prudently in patients with dyspnea and wheezing. Building on current guidelines for antibiotic treatment in children with febrile LRTI, a reliable decision algorithm for safe antibiotic withholding considering the laboratory and clinical factors evaluated in this study may have the potential to further reduce antibiotic prescribing.

## Supporting information

S1 Table Status classification for *Haemophilus influenzae* type b vaccination. (PDF)

S2 Table Status classification for *Pneumococcus* vaccination. (PDF)

S3 Table Reference ranges for white blood cell count (WBC) for university of basel children’s hospital, Switzerland. (PDF)

S4 Table Reference ranges for white blood cell count (WBC) for Kantonsspital Aarau, Switzerland. (PDF)

S5 Table Classification of tachypnea for age. (PDF)

S6 Table Classification of tachycardia for age. (PDF)

S7 Table Variables with significant associations antibiotic prescribing in univariate logistic analysis. (PDF)

S8 Table False positive and false negative prediction of antibiotic treatment by the multivariate logistic model. (PDF)

S9 Table Agreement of clinical and expert chest radiography diagnosis. (PDF)

S1 Fig Exploratory analysis for association of respiratory rate and heart rate with antibiotic prescribing. (PDF)

S2 Fig Transformation of C-reactive protein for logistic regression. (PDF)

S3 Fig Body temperature for logistic regression. (PDF)

S4 Fig Days of fever for logistic regression. (PDF)

S5 Fig Transformation of age for logistic regression. (PDF)

S1 Text Example codes of the main analyses. (PDF)
