# Supplementary material for: Drivers of antibiotic prescribing in children and adolescents with febrile lower respiratory tract infections
Source: PLoS One. 2017 Sep 28;12(9):e0185197. doi: 10.1371/journal.pone.0185197 (PMC5619731; doi:10.1371/journal.pone.0185197)
Supplement: S1 Table — (PDF) [file pone.0185197.s001.pdf]

S1 Table. Status classification for *Haemophilus influenzae* type B vaccination

Age (months)	complete	incomplete
0-3	all	-
>3-5	≥1x vaccinated	0x vaccinated or NA
>5-7	≥2x vaccinated	0-1x vaccinated or NA
>7-25	≥3x vaccinated	0-2x vaccinated or NA
>25	≥4x vaccinated	0-3x vaccinated or NA

The Swiss vaccination schedule was used as reference to classify the vaccination status into complete versus incomplete with respect to Haemophilus influenzae type B (Hib). At least four vaccinations are recommended (at 2, 4, 6, and 15-24 months). NA: Not applicable.
