# Supplementary material for: Drivers of antibiotic prescribing in children and adolescents with febrile lower respiratory tract infections
Source: PLoS One. 2017 Sep 28;12(9):e0185197. doi: 10.1371/journal.pone.0185197 (PMC5619731; doi:10.1371/journal.pone.0185197)
Supplement: S2 Table — (PDF) [file pone.0185197.s002.pdf]

S2 Table. Status classification for *Pneumococcus* vaccination

Age (months)	complete	incomplete
0-3	all	-
>3-5	≥1x vaccinated	0x vaccinated or NA
>5-13	≥2x vaccinated	0-1x vaccinated or NA
>13	≥3x vaccinated	0-2x vaccinated or NA

At least three vaccinations are recommended (at 2, 4 and 12 months). NA: Not applicable.
