# Supplementary material for: Drivers of antibiotic prescribing in children and adolescents with febrile lower respiratory tract infections
Source: PLoS One. 2017 Sep 28;12(9):e0185197. doi: 10.1371/journal.pone.0185197 (PMC5619731; doi:10.1371/journal.pone.0185197)
Supplement: S3 Table — (PDF) [file pone.0185197.s003.pdf]

S3 Table. Reference Ranges for White Blood Cell Count (WBC) for University of Basel Children's Hospital, Switzerland.

Age	Reported WBC normal range (G/L)
>1-12 months	≥6.0 to ≤17.5
>1-2 years	≥6.0 to ≤ 17.0
>2-4 years	≥5.5 to ≤ 15.5
>4-6 years	≥5.0 to ≤ 14.5
>6-10 years	≥4.5 to ≤ 13.5
>10-16 years	≥4.5 to ≤13.0
