# Supplementary material for: Drivers of antibiotic prescribing in children and adolescents with febrile lower respiratory tract infections
Source: PLoS One. 2017 Sep 28;12(9):e0185197. doi: 10.1371/journal.pone.0185197 (PMC5619731; doi:10.1371/journal.pone.0185197)
Supplement: S4 Table — (PDF) [file pone.0185197.s004.pdf]

S4 Table. Reference Ranges for White Blood Cell Count (WBC) for Kantonsspital Aarau, Switzerland.

**Age**	**Reported WBC normal range (G/L)**
>1-3 months	≥6.6 to ≤16.2
>3-12 months	≥6.6 to ≤15.6
>1-2 years	≥6.0 to ≤ 15.0
>2-4 years	≥5.4 to ≤ 13.8
>4-6 years	≥5.1 to ≤ 12.9
>6-12 years	≥4.8 to ≤ 12.0
>12-15 years	≥4.5 to ≤ 11.4
>15-18 years	≥4.5 to ≤ 10.8
