# Supplementary material for: Drivers of antibiotic prescribing in children and adolescents with febrile lower respiratory tract infections
Source: PLoS One. 2017 Sep 28;12(9):e0185197. doi: 10.1371/journal.pone.0185197 (PMC5619731; doi:10.1371/journal.pone.0185197)
Supplement: S5 Table — (PDF) [file pone.0185197.s005.pdf]

S5 Table. Classification of tachypnea for age.

Age range (WHO)	Upper limit (used for Classification)	Age range	Percentile 90[b] (1/min)
**< 2 months**	60/min[a]	< 2 months	60
**2-11 months**	50/min[a]	2-11 months	50
**1-5 years**	40/min[a]	1-2 years	40
		2-4 years	30
		4-10 years	25
		10-16 years	20

Since WHO did not provide reference values of respiratory frequency for children > 5 years, percentiles reported by Fleming et al. were used instead: [a]WHO; [b]Fleming et al. Lancet. 2011 March 19; 377(9770): 1011–1018
