# Supplementary material for: Drivers of antibiotic prescribing in children and adolescents with febrile lower respiratory tract infections
Source: PLoS One. 2017 Sep 28;12(9):e0185197. doi: 10.1371/journal.pone.0185197 (PMC5619731; doi:10.1371/journal.pone.0185197)
Supplement: S6 Table — (PDF) [file pone.0185197.s006.pdf]

S6 Table. Classification of tachycardia for age.

Age	Normal (1/min)	Reference range (1/min)[a]	Upper limit (used for classification) (1/min)
**1-11 months**	120	80-160	160
**1-2 years**	110	80-130	130
**>2-4 years**	100	80-120	120
**>4-6 years**	100	75-115	115
**>6-8 years**	90	70-110	110
**>10-12 years**	90	70-110	110
>12-14 years	90-85	70-110 (girls)<br>65-105 (boys)	110
>14-16 years	85-80	65-105 (girls)<br>60-100 (boys)	105

[a]Heart rate was classified according to: RE Behrman, HB Jenson and RM Kliegman. Nelson Textbook of Pediatrics, 17th edition 2004
