# Supplementary material for: Drivers of antibiotic prescribing in children and adolescents with febrile lower respiratory tract infections
Source: PLoS One. 2017 Sep 28;12(9):e0185197. doi: 10.1371/journal.pone.0185197 (PMC5619731; doi:10.1371/journal.pone.0185197)
Supplement: S7 Table — (PDF) [file pone.0185197.s007.pdf]

**S7 Table. Variables with significant associations antibiotic prescribing in univariate logistic analysis.**

| **Symptom/Variable**<br>(best threshold [95%CI], if appropriate) | **Sensitivity**<br><br>(95%CI) | **Specificity**<br><br>(95%CI) | **Accuracy**<br><br>(95%CI) | **AUC$_{ROC}$**<br><br>(95%CI) |
|---|---|---|---|---|
| Absence of wheezing at inclusion | 90% (84-96%) | 51% (40-63%) | 73% (68-73) | 0.71 (0.64-0.77) |
| C-reactive protein<br>(26 [12-55] mg/L) | 60% (50-70%) | 82% (72-90%) | 70% (63-76) | 0.80 (0.73-0.86) |
| WBC (outside reference range) | 50% (40-61%) | 88% (79-94%) | 67% (53-67) | 0.69 (0.63-0.76) |
| Age (2.1 [1.1-4.8] years) | 72% (62-81%) | 57% (46-69%) | 65% (58-73) | 0.67 (0.58-0.75) |
| Pneumoccoal vaccination<br>incomplete or unknown | 73% (63-82%) | 50% (39-61%) | 63% (56-70) | 0.62 (0.54-0.69) |
| Absence of dyspnea at inclusion | 49% (40-59%) | 79% (69-89%) | 62% (55-69) | 0.64 (0.57-0.71) |
| Negative or unknown RSV test | 83% (75-90%) | 35% (24-46%) | 62% (55-68) | 0.59 (0.52-0.66) |
| Elevated temperature<br>(38.6 [37.3-38.9] °C) | 50% (40-60%) | 76% (67-86%) | 62% (54-68) | 0.66 (0.58-0.75) |
| Preceding days of fever<br>(2.5 [1.5-5.5] days) | 63% (53-73%) | 58% (46-69%) | 60% (52-67) | 0.65 (0.57-0.73) |
| Reduced breathing sound | 40% (30-49%) | 86% (78-94%) | 60% (53-67) | 0.63 (0.57-0.69) |
| Pleuritic pain | 39% (29-48%) | 79% (69-88%) | 56% (50-63) | 0.59 (0.52-0.66) |

Sensitivity, specificity, accuracy and area under the receiver operating characteristics (ROC) curve (AUC$_{ROC}$) of variables showing significant associations in univariate logistic analysis.
