# Supplementary material for: Drivers of antibiotic prescribing in children and adolescents with febrile lower respiratory tract infections
Source: PLoS One. 2017 Sep 28;12(9):e0185197. doi: 10.1371/journal.pone.0185197 (PMC5619731; doi:10.1371/journal.pone.0185197)
Supplement: S8 Table — (PDF) [file pone.0185197.s008.pdf]

S8 Table. False Positive and False Negative Prediction of Antibiotic Treatment by the multivariate logistic model.

	**False positive classification**	**False negative classification**
Total number of patients [n]	17	15
Antibiotic prescription [n]<br>Day 1 > Day 1	 - (not applicable)	 10<br>5
Antibiotic pretreatment [n]	0	3
Diagnose [n]<br>Clinical at randomization<br>Bronchitis/Bronchiolitis<br>+ Pneumonia<br>Pneumonia<br>Chest radiograph (retrospective)<br>Bronchitis/Bronchiolitis<br>Bronchopneumonia<br>Lobar pneumonia<br>NA	 <br>9<br>5<br>3<br><br>10<br>2<br>0<br>5	 <br>4<br>5<br>6<br><br>8<br>6<br>0<br>1
CRP [mg/L (range)]	24 (4-144)	7.0 (3-36)
WBC [G/L (range)]	7.9 (4.4-24.4)<br>5 outside reference	9.9 (3.9-15.5)<br>3 outside reference
Temperature [°C (range)]	38.5 (36.9-39.9)	38.2 (36.7-39.9)
Preceding days of fever [days (range)]	3.0 (1-7)	4.0 (1-11)
Age [years (range)]	3.6 (0.1-14.4)	3.2 (0.4-9.4)
Heart rate [beats/min (range)]	136 (88-185)<br>(n=11 ↑ for age)	134 (92-152)<br>(n=8 ↑ for age)
Respiratory rate [breaths/min (range)]	32 (18-63)<br>(n=9 ↑ for age)	32 (20-60)<br>(n=10 ↑ for age)
Wheezing [n]	1	5
Dyspnea [n]	8	11
Reduced breathing sound [n]	5	3
Pleuritic pain [n]	2	3
Crackles[n]	7	3
Bronchial breathing [n]	4	3
Late inspiratory crackles [n]	8	8
Pleural rub [n]	0	0
Pneumococcal vaccination<br>[incomplete or unknown, n]	 11	 9
Hib vaccination<br>[incomplete or unknown, n]	 6	 3
NPA test [n]<br>All negative<br>RSV A/B positive<br>Influenza A/B positive<br>h-MPV<br>other	 5<br>5<br>3<br>2<br>2	 4<br>4<br>2<br>2<br>4

	(1x H1N1, 1x Influenza A + RSV A)	(1x H1N1, 2x M. pneumoniae, 1x C. pneumophilae + Influenza A)
Hospitalization [n]	7	8

NA: not applicable. CRP: C-reactive protein. WBC: White blood cell count. Hib: Haemophilus influenza type B. RSV: Respiratory syncytial virus. H-MPV: Human metapneumovirus. NPA: nasopharyngeal aspirate.
