# Supplementary material for: Drivers of antibiotic prescribing in children and adolescents with febrile lower respiratory tract infections
Source: PLoS One. 2017 Sep 28;12(9):e0185197. doi: 10.1371/journal.pone.0185197 (PMC5619731; doi:10.1371/journal.pone.0185197)
Supplement: S9 Table — (PDF) [file pone.0185197.s009.pdf]

S9 Table. Agreement of clinical and expert chest radiography diagnosis

		Emergency department Diagnosis (at study inclusion)		Agreement (missing data ignored)[c]	Agreement (missing data imputed)[d]
		**Bronchitis/ Bronchiolitis** (n=88, miss=32)	**Pneumonia** (n=201[a], miss=12)		
**Expert radiograph diagnosis (retrospective)**	**Bronchitis/ Bronchiolitis** (n=123)	57	66	46% (38-55%)	53% (45-61%)
	**Pneumonia** (n=166[b])	31	135	81% (75-87%)	78% (72-84%)
**Agreement (missing data ignored)[c]**		65% (55-75%)	67% (61-74%)	**Overall:** 66% (61-72%)	
**Agreement (missing data imputed)[d]**		67% (58-75%)	66% (60-73%)		**Overall:** 67% (61-72%)

a. 201 pneumonia patients = 124 cases of pneumonia + 77 cases of combined pneumonia and bronchitis/bronchiolitis; from n=12 pneumonia patients and n=32 bronchitis/bronchiolitis no radiograph was obtained (missing data)
b. 132 cases of bronchopneumonia + 34 cases of lobar pneumonia
c. proportion (exact binomial 95% confidence intervals)
d. pooled mean (95% confidence interval) of the 20 imputed datasets

Methods: The agreement of clinical and radiological diagnosis of pneumonia was evaluated in a contingency table, including both standard care control group and PCT -guided intervention group patients, and Cohen's Kappa statistic was calculated. Clinical diagnosis was made by the physicians at the emergency care units at study inclusion based on both clinical evaluation and radiographs, if available. Radiographs were later independently analyzed by a senior pediatric radiologist and a senior pediatric pulmonologist blinded to clinical or laboratory data. In case of disagreement, consensus was sought by discussion between the two raters. To assess the potential bias introduced by missing radiographs, 20 imputed datasets were created by fully conditional specification using the R package mice. The proportions of agreement were re-calculated for each of the imputed datasets and the pooled proportion and confidence interval were derived.

Results: Of 337 patients in both ProPAED study groups (intervention and control arm) 293 (87%) had radiographs available, of which 201 (69%) were diagnosed with CAP by pediatric emergency department physicians at study inclusion. WHO standardized assessment of chest radiographs classified 166 (57%) cases as CAP including 34 cases of lobar pneumonia. Clinical and standard WHO assessment agreed in 66% cases (Cohen's kappa = 0.29, 95%CI:

0.18-0.40, indicating “slight” to “fair” agreement). No bias was detected when reanalyzing the imputed data (mean agreement: 67%, Cohen’s kappa: 0.30).
