# Supplementary material for: Drivers of antibiotic prescribing in children and adolescents with febrile lower respiratory tract infections
Source: PLoS One. 2017 Sep 28;12(9):e0185197. doi: 10.1371/journal.pone.0185197 (PMC5619731; doi:10.1371/journal.pone.0185197)
Supplement: S1 Fig — (PDF) [file pone.0185197.s010.pdf]

S1 Fig. Exploratory Analysis for Association of Respiratory Rate and Heart Rate with Antibiotic Prescribing

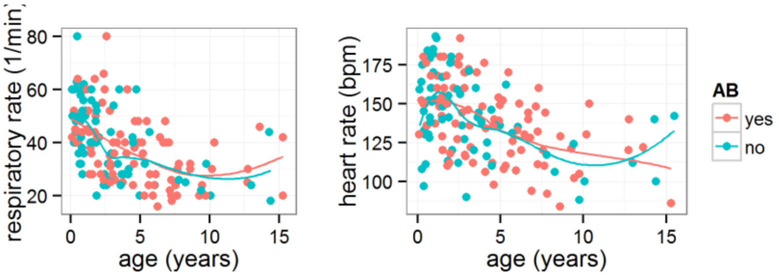

Although no association between age-dependent respiratory rate (breath rate, BR) or heart rate (HR) and antibiotic prescribing could be seen in the exploratory analysis, variables indicating high HR and BR for age were created according to S5 Table.
