# Supplementary material for: Drivers of antibiotic prescribing in children and adolescents with febrile lower respiratory tract infections
Source: PLoS One. 2017 Sep 28;12(9):e0185197. doi: 10.1371/journal.pone.0185197 (PMC5619731; doi:10.1371/journal.pone.0185197)
Supplement: S2 Fig — (PDF) [file pone.0185197.s011.pdf]

S2 Fig. Transformation of C-reactive Protein for Logistic Regression.

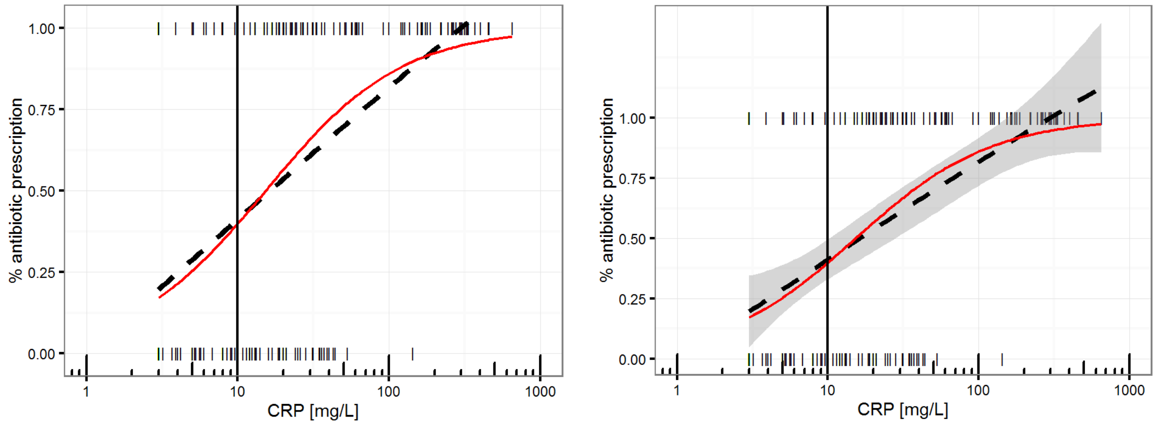

Observed antibiotic prescription according to CRP values (|) versus predicted probability (red line) of antibiotic prescription after log-transformation of CRP ($\log_{10}$ was used for better interpretability of regression coefficients). As reference value 10 mg/L was chosen (instead of 1 mg/L which would be the default reference value using a log-transformation). Dashed line: non-parametric regression line. The confidence interval (shaded area) of this non-parametric regression line is indicated separately in the right panel, since it may comprise values >1 or <0. Black vertical line: Reference value (10 mg/L) corresponding to the intercept of the estimated logistic regression model.
