# Supplementary material for: Drivers of antibiotic prescribing in children and adolescents with febrile lower respiratory tract infections
Source: PLoS One. 2017 Sep 28;12(9):e0185197. doi: 10.1371/journal.pone.0185197 (PMC5619731; doi:10.1371/journal.pone.0185197)
Supplement: S3 Fig — (PDF) [file pone.0185197.s012.pdf]

S3 Fig. Body Temperature for Logistic Regression.

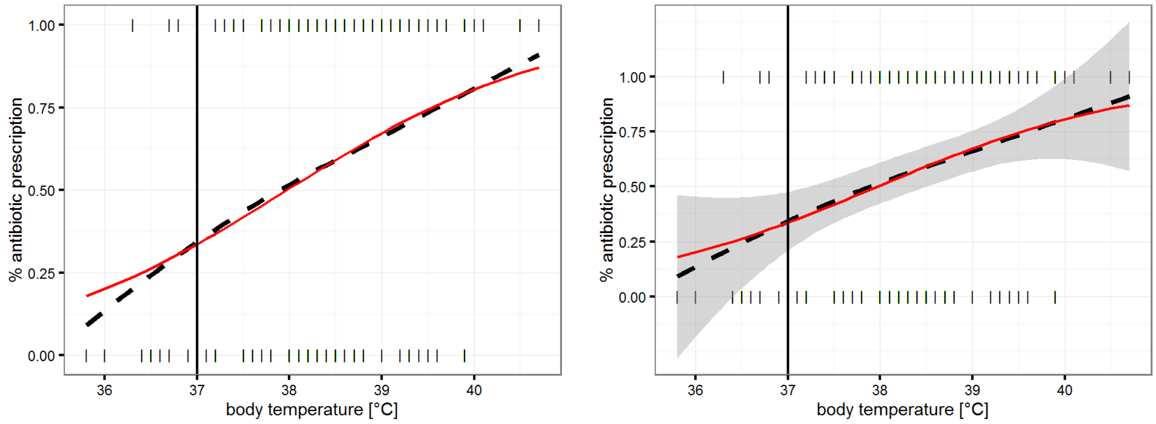

The relationship between body temperature and antibiotic prescription was well described by a simple linear relationship in the logistic regression without the need for transformation. Observed antibiotic prescribing according to body temperature (|) versus predicted probability (red line) of antibiotic prescription. Dashed line: non-parametric regression line. The confidence interval (shaded area) of this non-parametric regression line is indicated separately in the right panel, since it may comprise values >1 or <0. Black vertical line: Reference value (37°C) corresponding to the intercept of the estimated logistic regression model.
