# Supplementary material for: Drivers of antibiotic prescribing in children and adolescents with febrile lower respiratory tract infections
Source: PLoS One. 2017 Sep 28;12(9):e0185197. doi: 10.1371/journal.pone.0185197 (PMC5619731; doi:10.1371/journal.pone.0185197)
Supplement: S5 Fig — (PDF) [file pone.0185197.s014.pdf]

S5 Fig. Transformation of Age for Logistic Regression.

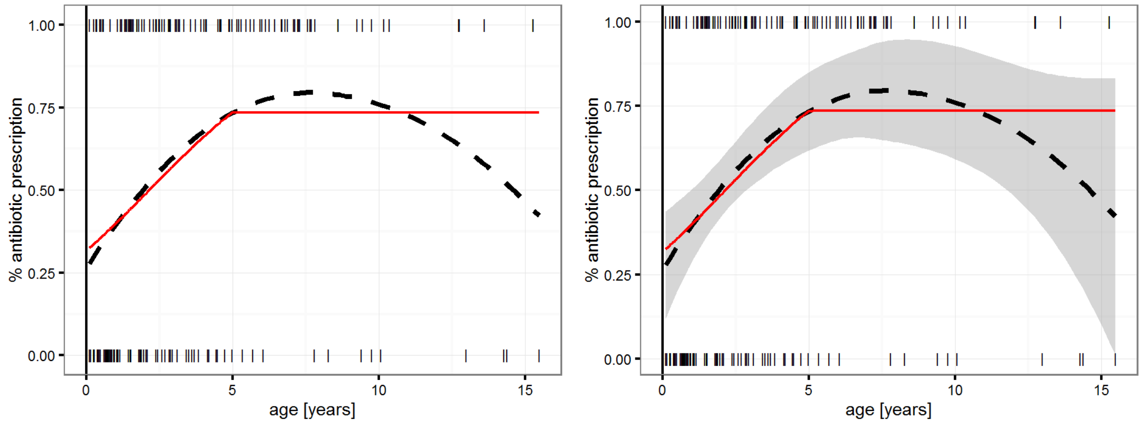

Antibiotic prescription according to the age of patient after a piece-wise linear function (increase in frequency of antibiotic prescription during the first 5 years of life, after 5 years no further increase is observed) (|) versus predicted probability (red line) of antibiotic prescription. Dashed line: non-parametric regression line. The confidence interval (shaded area) of this non-parametric regression line is indicated separately in the right panel, since it may comprise values >1 or <0. Black vertical line: Reference value corresponding to the intercept of the estimated logistic regression model.
