# Supplementary material for: Drivers of antibiotic prescribing in children and adolescents with febrile lower respiratory tract infections
Source: PLoS One. 2017 Sep 28;12(9):e0185197. doi: 10.1371/journal.pone.0185197 (PMC5619731; doi:10.1371/journal.pone.0185197)
Supplement: S1 Text — (PDF) [file pone.0185197.s015.pdf]

S1 Text
Example codes of the main analyses

Univariate regression

```
#------------------------
# Data transformation
#------------------------
# Transformation of continuous variables to linearize relationships and centre baseline to more
meaningful value
# example: CRP (lab result in mg/dL) was log10-transformed and centered to 10 mg/dL  -> new
variable lCRP10

data <- within(data, {
lCRP10 <- log10(CRP/10)
x1 <- lCRP10
})

#------------------------
# fit logistic regression model,
#------------------------
# evaluate linear relationship between variable x1 and antibiotic prescription y (0/1)

unimod1 <- glm(y ~ x1, family="binomial", data=data)

#------------------------
# estimates and 95% CI
#------------------------

# estimated coefficients, standard errors, z- and p-values
sum1 <- summary(unimod1)$coefficients

# extract effect estimates and standard error
est <- sum1["x1","Estimate"]
est.se <- sum1["x1","Std.Error"]

# extract baseline estimates and standard error
bl <- sum1["x1","Estimate"]
bl.se <- sum1["x1", "Std.Error"]

# functions to calculate probability (prob) and odds ratios (OR)
# from logistic regression coefficients
prob <- function(a, b) 1/(1+exp(-(a+b))) # a=intercept, b=estimated covariate effect
OR <- function(c) exp(c) # c=estimated covariate effect

# calculate OR with 95% CI (ll= lower limit, ul=upper limit)
OR.est = OR(est)
OR.ll = OR(est-1.96*est.se)
OR.ul = OR(est+1.96*est.se)

# calculate basline probablitly with 95% CI
PBL = prob(a=bl, b=0)
PBL.ll = prob(a=bl-1.96*bl.se, b=0)
PBL.ul = prob(a=bl+1.96*bl.se, b=0)
```

Univariate classification & ROC-analysis

```
library(pROC)

# ROC analysis
roc1 <- roc(y~x1, ci=TRUE)

# obtain AUC estimate with 95% CI
roc1

# estimate best threshold for continuous covariate
thresh1 <- ci.coords(roc1, x="best", ret=c("threshold"))

# CI of sensitivity, specificity and accuracy using estimated best threshold
coordsp1 <- ci.coords(roc1, x=thresh1[2], ret=c("specificity", "sensitivity", "accuracy"))
```

Multivariate regression & model evaluation

```
# fit multivariate logistic regression model, in this example using 3 linear predictor variables
(x1, x2, x3)
mfinal <- glm(y ~ x1 + x2 + x3, family="binomial")

# summary of estimates, OR, and probabilities can be calculated as shown above from
summary(mfinal)

#------------------------
# Model evaluation
#------------------------

# global goodness of fit
library(rms)
# lrm = logistic regression model using package rms
mfinal <- lrm(y ~ x1 + x2 + x3, x=TRUE, y=TRUE, method="lrm.fit", linear.predictors=TRUE,
se.fit=FALSE)
# Cessie - van Houwelingen - Copas - Hosmer unweighted sum of squares test for global goodness
of fit
residuals(mfinal, type="gof")

# (over)dispersion
# Pearson residuals
res <- residuals(mfinal, type="pearson)

# dispersion parameter sigma2
sigma2 <- sum(res^2)/length(res)

# residuals
library(faraway)
halfnorm(residuals(mfinal))
plot(predict(mfinal, type="link"), residuals(mfinal, type="pearson"))

# single term deletion
drop1(multi5, scale=sigma2, test="LRT") # with estimated dispersion

#-------------------------------------------------------------------
# predictive performance
#----------------------------------------------------------------------

# using a threshold of predicted probability P=0.5, i.e.
# if P >= 0.5 => prediction = 1, i.e. antibiotic prescription = yes;
# if P < 0.5 => prediction = 0, i.e. antibiotic prescription = no)

threshold <- 0.5

# model-predicted antibiotic prescription (TRUE or FALSE) for each observation
class <- predict(mfinal, type="response")>=threshold

# contingency table
tab <- table(class, mfinal$y)

# calculate performance measures with binomial 95% CI
sensitivity <- binconf(tab["TRUE","1"], sum(tab[,"1"]), method="exact")
specificity <- binconf(tab["FALSE","0"], sum(tab[,"0"]), method="exact")
accuracy <- binconf(tab["TRUE","1"]+tab["FALSE","0"], sum(tab), method="exact")
```

Sensitivity analyses

1) Interaction / subgroup analysis for pleuritic pain in patients aged >/< 2 years

```
# create new factor "fage2" (>/< 2 years) from variable "age"
fage2 <- cut(age, c(0, 2, 18))

# interaction model
glm(y~pleupain*fage2, family="binomial")

# subgroup analysis
glm(y~pleupain, family="binomial", data= subset(data, age<=2))
glm(y~pleupain, family="binomial", data= subset(data, age>2))
```

2) Elastic net regression

```
library(glmnet)
y <- data$y

# matrix of possible predictor variables
x <- as.matrix(data[,2:ncol(data)])

set.seed(123) # set seed for cross-validation

# fit generalized linear model via penalized maximum likelihood and determine optimal lambda
using 100-fold cross-validation (cv) for lowest mis-classification error

# (1) using alpha = 0.5 -> elastic net regression
cvfit_class = cv.glmnet(x, y, family="binomial", standardize = TRUE, alpha=0.5,
type.measure="class", nfold=100)
# illustrate
plot(cvfit_class)
# optimal lambda (lowest mis-classification error)
coef(cvfit_class, s="lambda.min")
# accepting larger error, but within 1 standard error
coef(cvfit_class, s="lambda.1se")

# (2) using alpha = 1 -> lasso
cvfit_class = cv.glmnet(x, y, family="binomial", standardize = TRUE, alpha=1,
type.measure="class", nfold=100)
plot(cvfit_class)
coef(cvfit_class, s="lambda.min")
```

3) Imputation of missing data using fully conditional specification

```
library(mice)

# make an initial mice object to adapt predictors and methods:
ini <- mice(mytransdat, max=0, print=FALSE)
# predictor matrix
mypred <- ini$predictorMatrix

# derived variables (e.g. variable "lCRP10" should not be used as predictor
mypred[,"lCRP10"] <- 0

# impuation methods
```

```
mymeth <- ini$meth
# used methods: "logreg" for binary variables, "ppm" for continuous variables, "polyreg" for
categorical with > 2 levels

# passive impution for transformed variables - keep transformations, e.g.
mymeth["lCRP10"] <- "~(log10(CRP/10))"

# 20 imputations of data with missing variables
imp20 <- mice(data, m=20, pred=mypred, method=mymeth)

# re-fit regression model and summarize pooled estimates of n=20 regression models
fit1 <- with(imp20, glm(y~x1, familiy="binomial")
pool(fit1)
```
